# Polyvinylimidazole-Based Cryogel as an Efficient Tool for the Capture and Release of Oleuropein in Aqueous Media

**DOI:** 10.3390/polym16162339

**Published:** 2024-08-18

**Authors:** Valentina Giglio, Chiara Zagni, Emanuela Teresa Agata Spina, Francesca Cunsolo, Sabrina Carola Carroccio

**Affiliations:** 1 CNR—Institute of Biomolecular Chemistry, Via Paolo Gaifami 18, 95126 Catania, Italy; francesca.cunsolo@icb.cnr.it; 2 Department of Drug and Health Sciences, University of Catania, V.le A. Doria 6, 95125 Catania, Italy; chiara.zagni@unict.it; 3 CNR—Institute for Polymers, Composites and Biomaterials, Via Paolo Gaifami 18, 95126 Catania, Italy; emanuelateresaagata.spina@cnr.it (E.T.A.S.); sabrinacarola.carroccio@cnr.it (S.C.C.)

**Keywords:** recovery, hydrogel, adsorption, polymer, oleuropein, imidazole

## Abstract

A polyvinylimidazole-based cryogel is presented as a pioneering solution for efficient extraction and release of partially water-soluble polyphenols from olive byproducts. Specifically, oleuropein was used as model molecule to evaluate its recovery from water. The material merges the properties of interconnected cryogel structure in adsorbing molecules via fast diffusion flux, with the strong electrostatic interactions acted by imidazole moiety. Such cryogel achieves effective oleuropein binding likely through hydrogen bonding and π–π interactions. Comprehensive assessments of static adsorption kinetics, isotherms, and desorption kinetics underscore the cryogel’s efficacy in oleuropein extraction and release, highlighting its pivotal role in valorizing olive wastewater through sustainable biotechnological applications.

## 1. Introduction

Oleuropein, a phenolic compound found in olive leaves, olive oil, and olive byproducts, has emerged as a valuable substance within the olive industry. Traditionally considered a waste product, oleuropein is abundant in the leaves and other residual materials left after olive oil production. As a key constituent of the olive tree (Olea europaea), oleuropein has been extensively studied for its diverse range of biological activities and health benefits [1].

This compound belongs to the secoiridoid group of phenolic derivatives and is known for its powerful antioxidant properties, which help to protect cells from oxidative damage. Additionally, oleuropein exhibits anti-inflammatory, antimicrobial, and antiviral activities, making it a compound of significant interest in the fields of nutrition and medicine. Its multifaceted benefits contribute to the health-promoting qualities of the Mediterranean diet, renowned for its association with longevity and reduced incidence of chronic diseases [2]. The possible utilization of oleuropein extracted from olive industry byproducts could not only promote sustainability by reducing waste but also open new avenues for developing natural health supplements and functional foods.

Efficiently extracting oleuropein from olive leaves presents a significant challenge due to the complex nature of the extraction process and the variability of oleuropein content in the raw material. Moreover, during the separation process, several parameters, such as extraction solvent composition, temperature and pH, and the type of extraction method, can highly affect the recovery of oleuropein [3]. Recently, a green chemistry approach using non-petroleum-derived solvents has been employed in the extraction of phenolic substances to promote sustainable processes [4,5]. Among them, glycerol has lately been suggested as a non-toxic solvent for polyphenols extraction, but efficient oleuropein purification has been obtained only when cyclodextrin was added to the solvent medium [6,7]. However, the encapsulation technique can be a complex and expensive process, potentially increasing costs and impacting the commercial success of the extracts.

Ionic liquids (ILs) are organic salts liquid below 100 °C, known for their high thermal stability, low volatility, and excellent solvation ability. Their physicochemical properties can be tailored by modifying their cation or anion structures, making them ideal for specific processes [8]. ILs are widely used in extraction, separation, organic synthesis, and more. ILs are excellent extractors of molecules due to their unique interactions, such as cation–anion interactions, hydrogen-bonding, and hydrophobic interactions [9,10]. These interactions enhance the solubility of natural products and selectivity of their extraction. To leverage these advantages, Yi Liu et al. integrated imidazole in the form of ILs into a polymeric material to exploit the adsorbing effect of the polymer matrix [11]. This approach aims to simplify handling, enable controlled release and recovery, and ensure efficient extraction. Importantly, this approach offers environmental benefits by promoting greener and more sustainable processes. Such polymeric ionic liquids effectively captured oleuropein by electrostatic, hydrogen, and stacking interactions between the imidazole moiety and oleuropein. However, with this approach, the content of oleuropein adsorbed per unit weight of the polymer (Qe) did not exceed 20 mg g^−1^ of material. This limitation is likely attributed to the low amount of imidazole in the system, since it was grafted to the polymer in a second stage, rather than during the synthetic process.Relevant results were achieved with macroporous adsorption resins (MARs), widely used to separate and purify plant secondary metabolites, such as flavonoids and alkaloids, from crude herbal extracts [12,13,14,15,16]. Recently, boric acid affinity resin as well as carboxyl-modified multiwalled carbon nanotube resin, have been used for oleuropein isolation and purification [17,18]. In particular, Liu et al.’s approach, combined with a mixed solvent composed of ethyl acetate, ethanol, and water, allowed for a more than fivefold increase in the separation and purification of oleuropein.

Cryogels are macroporous materials known for their large, interconnected pores, making them ideal for the rapid and selective extraction of target molecules from complex aqueous mixtures. Their robustness, flexibility, and customizable properties enable various applications, including contaminant removal, biomolecule purification, and active pharmaceutical ingredient isolation [19,20,21,22,23,24,25].

The ability to engineer cryogels with specific pore sizes and functional groups enhances their affinity and selectivity, making them invaluable in environmental, biotechnological, and pharmaceutical applications.

In particular, imidazole-based cryogels have proven to be highly effective systems for capturing metals such as copper [26], cobalt, and nickel [27]. Additionally, in their protonated forms, they have demonstrated efficiency in capturing anionic pollutants like methyl orange [28]. These studies underscore the remarkable versatility of these materials.

Within this framework, this study aims to combine the previously reported advantages of imidazole and polymeric materials to produce a highly efficient tool for capturing and releasing oleuropein as well as other water-soluble polyphenols from aqueous media.

The novelty of this work lies in the application of a cryogel composed entirely of imidazole units which proves to be particularly effective in capturing and recovering oleuropein. This application has not been explored with the previously reported cryogels, highlighting the potential of the synthesized material in the efficient recovery of this valuable compound. Our system provides a low-cost and simple method for capturing oleuropein, addressing a significant gap in the current literature.

By leveraging the unique adsorbent properties of porous cryogels enhanced by the imidazole moiety, we demonstrate a significant advancement in the functional use of polyvinylimidazole-based materials. In this regard, the imidazole groups facilitate effective binding through hydrogen and π–π interactions, leading to superior performance in polyphenol capture. Static adsorption kinetics, isotherms, and desorption kinetics were thoroughly investigated as part of this study.

## 2. Materials and Methods

### 2.1. Materials

*N*-vinylimidazole (VI), *N*,*N*′-methylenebisacrylamide (MBAA), ammonium persulfate (APS), *N*,*N*,*N*′,*N*′-tetramethylene diamine (TEMED), and oleuropein commercial standard (Oleo) were purchased from Merck (Milan, Italy) and used without further treatments. A Milli-Q water purification system produced deionized water.

### 2.2. Synthesis of Poly(VI) Cryogel

The poly(VI) cryogel was prepared via free radical polymerization initiated by APS and TEMED according to the cryogelation method [29,30,31]. In brief, 1754 μL of H_2_O, 100 mg of *N*-vinylimidazole (VI) and 23 mg of *N*,*N*’-methylenebisacrylamide (MBAA, molar ratio 1/6 compared to the moles of VI) were mixed in a 4 mL vial. Then, the solution was cooled to 0 °C, followed by the addition of 25 μL each of a 10% *w*/*v* solution of ammonium persulphate (APS) and tetramethylethylenediamine (TEMED) in water, under vigorous stirring. After the addition of APS and TEMED at 0 °C, the solution was mixed for only 1 min before transferring the reaction mixture into a 5 mL syringe pre-cooled to 0 °C and then placed in a cryostat at −15 °C for about 24 h. Then, after thawing, the cryogel obtained was washed initially with H_2_O (50 mL), followed by a stepwise wash with a mixture of H_2_O–EtOH (50 mL), progressively increasing the EtOH concentration up 100% (50 mL) and dried in a vacuum (−1 bar) oven at 40 °C over night. The final product consists of a macroporous monolithic cryogel. The yield of reaction ranges from 80 to 85%.

### 2.3. Cryogel Characterization

Poly(VI) cryogel underwent characterization using various techniques to assess their physicochemical and morphological properties.

Thermal behaviors and kinetics parameters of the synthesized cryogel were determined using a PerkinElmer thermogravimetric apparatus (TGA). The TGA sensitivity was 0.1 μg with a weighting precision of ±0.01%. The measurements were performed in a temperature range of 40 °C to 700 °C, at a heating rate of 10 °C min^−1^ under nitrogen flow (60 mL min^−1^), using 2 ± 0.1 mg of sample.

ATR FT-IR spectra were acquired in the 4000–400 cm^−1^ region on PerkinElmer Spectrum Two FT-IR spectrometer equipped with an internal reflection crystal of zinc selenide (ZnSe). For each spectrum, 32 scans at a resolution of 4 cm^−1^ were collected.

Surface morphologies of the cryogel sample were observed using a desktop scanning electron microscopy (SEM) Thermo Phenom Prox system (Thermo Fisher Scientific—Waltham, MA, USA) combined with a fully integrated energy-dispersive X-ray detector (silicon drift detector).

Swelling tests were performed on dried cylindric samples of about 50 mg. After 30 s of water uptake and removal of its excess from the surface, mass measurements were performed. Each sample was weighed three times (Table 1):

The total water uptake was determined by calculating the increase in mass, using Equation (1):

(1) $$S\%=\frac{W_{swollen}-W_{dry}}{W_{dry}}\times 100$$

where *W_swollen_* represents the weight of the sample after solvent uptake, and *W_dry_* is the dried weight.

### 2.4. Adsorption Kinetics of Oleuropein

For kinetics studies, 0.010 g of dried cryogel was immersed into 2 mL of oleuropein aqueous solution at a concentration of 50 ppm. The solution was shaken at a speed of 200 rpm at room temperature for a specific time. The adsorption capacity *Q_t_* (mg g^−1^) of the cryogel at different time intervals was calculated as follows, Equation (2):

(2) $$Q_{t}=\frac{\left(C_{0}-C_{t}\right)\times V}{m}$$

where *C*_0_ and *C_t_* are the initial and t-time concentration of oleuropein in solution, respectively; *V* (L) and *m* (g) are the volume of solution and the mass of the cryogel used, respectively. The *C*_0_ and *C_t_* were determined using a UV–vis JASCO spectrophotometer measuring the absorbance at 232 nm at different times. In order to investigate the mechanism of the adsorption process, pseudo-first-order (Equations (3) and (5)) and pseudo-second-order (Equations (4) and (6)) models fit were applied to the experimental data, according to the equations below. In particular, both linear and non-linear models were used to describe the kinetics curves.

For non-linear pseudo first and pseudo-second order model fitting were used Equations (3) and (4), respectively:

(3) $$Q_{t\;}=\;Q_{e}\times (1-e^{-k_{1}t})\;\;\;$$

(4) $$Q_{t\;}=\frac{K_{2}Q_{e}^{2}t}{1+K_{2}Q_{e}t}$$

The corresponding linearized equation form of pseudo-first- and pseudo-second-order used were, respectively, Equations (5) and (6):

(5) $$\ln\left(Q_{e}-Q_{t}\right)=\ln Q_{e}-K_{1}kt\;$$

(6) $$\frac{t}{Q_{t}}=\frac{1}{k_{2}Q_{e}^{2}}+\frac{1}{Q_{e}}t$$

where *Q_e_* (mg g^−1^) and *Q_t_* (mg g^−1^) are the oleuropein adsorption capacity of cryogels at equilibrium and at time *t* (min), respectively; *k*_1_ (L min^−1^) and *k*_2_ (g/(mg min^−1^) are rate constants of the pseudo-first-order and pseudo-second-order, respectively.

### 2.5. Adsorption Isotherm of Oleuropein 

Adsorption isotherm studies were performed by adding 0.010 g of dried poly(VI) cryogels into 2 mL of oleuropein aqueous solution at desired concentrations (50–300–500–1000 ppm), followed by shaking gently for 24 h to ensure that equilibrium was reached. The static Oleo-adsorption capacities of the cryogel for each concentration of Oleo solution used were calculated according to Equation (7):

(7) $$Q_{e}=\frac{\left(C_{0}-C_{e}\right)\times V}{m}$$

where *C*_0_ (mg L^−1^) is the initial concentration of oleuropein for each batch, *C_e_* (mg L^−1^) is the concentration at the equilibrium, *V* (L) is the volume of water, and *m* (g) is the weight of adsorbent used during the adsorption experiments.

Langmuir and Freundlich models were used to fit the adsorption data according to the following Equations (8) and (9), respectively:

(8) $$Q_{e}=\frac{Q_{max}K_{L}C_{e}}{\left(1+K_{L}C_{e}\right)}$$

where *Q_e_* (mg g^−1^) is the content of Oleo adsorbed per unit weight of the polymer, *C_e_* (mg L^−1^) is the Oleo concentration at the equilibrium, *Q_max_* (mg g^−1^) is the monolayer capacity, and *K_L_* (L mg^−1^) represents the Langmuir constant.

(9) $$Q_{e}=K_{F}C_{e}^{1/n}$$

where *Q_e_* (mg g^−1^) is the content of Oleo adsorbed per unit weight of polymer, *C_e_* (mg L^−1^) is the Oleo concentration at the equilibrium, *K_F_* (mg g^−1^) represents the Freundlich constant, and 1/*n* is a parameter that allows obtaining indications about the heterogeneity of the system.

The corresponding linearized equation form of Langmuir and Freundlich model used were Equations (10) and (11), respectively:

(10) $$\frac{1}{Q_{e}}=\frac{1}{k_{L}q_{m}}\times \frac{1}{C_{e}}+\frac{1}{Q_{max}}$$

(11) $$lnQ_{e}=\frac{1}{n}lnc_{e}+lnk_{F}$$

### 2.6. Release of Oleuropein and Reusability of Poly(VI)

The release of Oleo was tested in two different media: an ethanolic solution at 70% and a saline buffer consisted of a ready solution of PBS (phosphate-buffered saline, pH 7.4). 0.010 g of poly(VI) loaded with a solution of Oleo (50 ppm) was placed in a vial with 2 mL of solution (ethanolic and saline). The released amount of Oleo was determined using a UV–vis JASCO spectrophotometer measuring the absorbance at 232 nm at different times: 20, 100, 200, 300, 500, 600, 720 min.

To test the reusability of the poly(VI) cryogel, five consecutive cycles of adsorption and desorption were performed. To evaluate the complete regeneration of the material, after each cycle of desorption the cryogel was washed thoroughly with distilled water to ensure removing salt, then washed with ethanol, and finally dried before performing the next step of adsorption.

## 3. Results

### 3.1. Synthesis and Characterization of the Material

**Figure 1:**
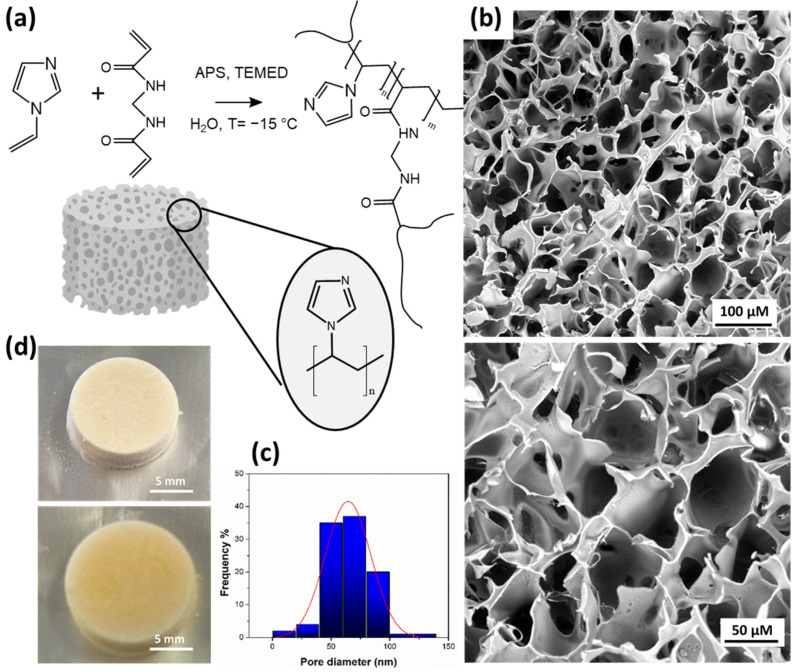
(**a**) Scheme of reaction for the synthesis of poly(VI); (**b**) SEM images of poly(VI); (**c**) pore diameter distribution; (**d**) pictures of poly(VI) before and after water uptake.

The poly(VI) cryogel was prepared by the co-polymerization of VI (monomer) with MBAA (cross-linker) according to the chemical reaction shown in Figure 1a. The formation of the homogeneous macroporous structure in the poly(VI) cryogel is evidenced by SEM images (Figure 1b), which reveal macropores ranging in diameter from 20 to 150 μm (Figure 1c). This structural characterization underscores the effectiveness of the cryogelation process in achieving a well-defined and interconnected network of pores. This morphology facilitates rapid mass transfer allowing to obtain high flux in the practice application for the 3D monolithic cryogel. Furthermore, the large pore size distribution observed in poly(VI) can positively influence the fast diffusion of water media into the interconnected system, as shown by swelling capacity tests performed on the dried sample, Figure 1d [32]. This results in a higher number of VI-active sites becoming available in a short period, thereby improving the capture oleuropein in solution.

**Figure 2:**
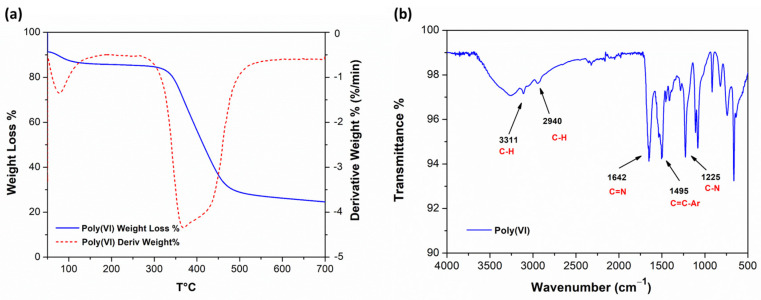
(**a**) TGA and DTGA of poly(VI); (**b**) ATR–FTIR spectrum of poly(VI).

The thermal behavior of poly(VI) was investigated by means of thermogravimetric analysis under inert conditions. Figure 2a shows the TGA and DTGA profiles of poly(VI) cryogel; the sample loses water at 50 °C. This first stage of weight loss was completed at 100 °C with about a 6% decrease in weight. The second stage of thermal degradation started from ∼300 °C to ∼450 °C, with a maximum weight loss at 370 °C. Upon conclusion of the thermal treatment at 700 °C, the mass percentage residual is 24%; this result is not trivial because the char formation due to the high aromatic content of the polymeric material under inert conditions [19,28].

The ATR–FTIR characterization of poly(VI) is reported in Figure 2b. The spectrum shows the characteristic peak at 3311 cm^−1^ for C–H stretching, the 1498 cm^−1^ stretching peaks for C=C. Additionally, the peaks at 1642 cm^−1^ and 1225 cm^−1^ are related to C=N and C–N stretching, respectively. These FT-IR peaks are also present in the VI monomer, as reported in the literature [27]. Additional peaks are 1114 cm^−1^ for ring vibration, 1088 cm^−1^ for in-plane ring C–H bending, and 911 cm^−1^ for ring deformation [26]. The new band at 2940 cm^−1^ was assigned to the C–H-stretching vibration of the newly formed polymer chain.

The swelling capacity of poly(VI) in aqueous solution was also assessed. In Figure 1d, the pictures of the cryogel before and after water uptake are shown; as can be seen from the pictures, the change in volume is not as significant as that observed in other sponge materials, likely due to the structural rigidity conferred by imidazole. Nevertheless, the high-interconnected porous surface area allows for the rapid diffusion of water, achieving a swelling degree of about 310 ± 10% in few seconds. The latter data are consistent with the findings reported in the literature for similar materials [27,28] and, as expected, reflect a certain degree of hydrophobicity of the polymer.

### 3.2. Adsorption Kinetics Analysis

Adsorption kinetics analyses show the absorption feature of an adsorbent material for its usage in common practice applications by describing its adsorption rate and adsorption efficiency. These studies were performed in accordance with the experimental conditions reported in Section 2.4.

**Figure 3:**
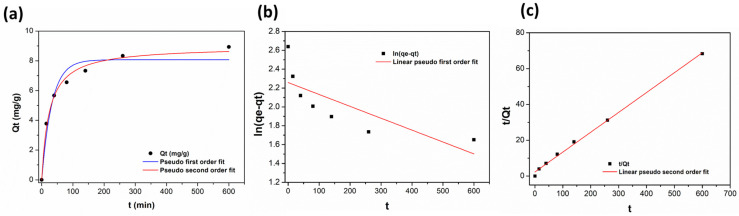
(**a**) Adsorption kinetics of Oleo on poly(VI) at 25 °C. (**b**) Linearized pseudo-first-order fitting of experimental data; (**c**) linearized pseudo-second-order fitting of experimental data.

Experimental kinetics adsorption of a standard solution of Oleo (50 ppm) on poly(VI) cryogel at room temperature is shown in Figure 3a. It can be observed that the adsorption capacities of polymer increased as contact time reached a plateau at 600 min. The experimental points can be divided into two regions (I and II). In region I, the uptake (*Q_t_*) of oleuropein increased rapidly, thanks to the large, interconnected porous structure of the material, which allows a fast diffusion of the oleuropein solution favoring its contact with the binding site, assuring low mass transfer resistance in the bulk solution. After 100 min of contact time (region II), the experimental absorption rate evolves slowly probably because the pores and the absorption site of outer surface are already occupied. The *Q_t_* values increased gradually and became almost constant around a value of 9 mg g^−1^ (corresponding to the *Q_e_* value for the starting Oleo solution of 50 ppm).

To better understand the adsorption mechanism, the pseudo-first-order and pseudo-second-order kinetics equation were applied to the experimental data. Figure 3 shows the two model fittings applied in their nonlinear form (Figure 3a) and linearized forms (Figure 3b,c). The model that best fit the data was the pseudo-second-order, the choice was made based on the correlation coefficient R^2^ and other kinetics parameter reported in Table 2. Actually, from the results obtained with a non-linear-type fitting both kinetic models could be used to fit the first part of kinetics process, but, after 100 min, the model that better described the adsorption mechanism was the pseudo-second-order. Our data are in agreement with similar adsorbed systems [19]. The R^2^ of the pseudo-second-order model has a value of 0.99, denoting that this model well describes the experimental data. Moreover, the value of *Q_e_* obtained was pretty close to the experimental one, *Q_ecalc_* = 8.96 vs. *Q_eexp_* = 8.77, thus reinforcing the goodness of the model, highlighting that the adsorption process is mainly controlled by chemisorption, due to the pivotal role of hydrogen-bonding and π–π interactions provided by imidazole moiety [33]. On the other hand, an R^2^ of 0.94 was obtained when a non-linear equation of the pseudo-first-order model was applied; however, the linearization of this model lowers the R^2^ to 0.54, indicating that this model is not fully applicable to this experimental data set.

### 3.3. Adsorption Isotherms Analysis

**Figure 4:**
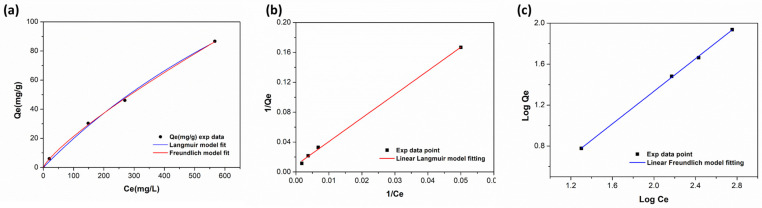
(**a**) Equilibrium adsorption isotherm of Oleo on poly(VI); (**b**) linearized Langmuir model fitting of experimental data; (**c**) linearized Freundlich model fitting of experimental data.

To evaluate the absorption capacity of poly(VI), different oleuropein aqueous solutions, ranging from 50 to 1000 ppm, were prepared and incubated with a fixed amount of polymeric adsorbent (0.010 g) until the adsorption equilibrium was reached. The absorption capacity (*Q_e_*) was calculated for each solution, following Equation (7), and the experimental data obtained are reported in Figure 4.

As expected, the adsorption capacities increased with an increasing concentration of oleuropein in solution, indicating that higher concentrations lead to greater equilibrium adsorption capacities.

To explore the mechanism of adsorption processes, the experimental data were fitted using Langmuir and Freundlich isotherm models. The Langmuir equation (Equation (8)) is one of the most important isotherms adsorption models to obtain useful information about the adsorbate/adsorbent interaction. In the Langmuir model, it is assumed that the adsorption process occurs at homogeneous sites inside the adsorbent and that these adsorption sites have the same energy; so, this model is frequently applied to homogenous systems.

The Freundlich sorption model is usually used to describe heterogeneous systems (Equation (9)). The adsorption process, in this instance, is not limited to the creation of an adsorbate monolayer; additionally, distinct adsorption sites on the adsorbent surface display varying affinities for the adsorbate. Considering Equation (9), the parameter that best describes this phenomenon is 1/*n*, where *n* is an indicator of how the adsorbent’s affinity for the adsorbate changes in response to the adsorption density modifications. The closer 1/*n* gets to 0, the more heterogeneous the adsorbent surface becomes, while when the value of 1/*n* above 1, it is indicative of cooperative adsorption [11]. In our case, the value of 1/*n* obtained was close to 1 (see Table 3), indicating that the adsorption was a favorable physical adsorption process.

For the calculation of the isotherm parameters, the linearized form equation for Langmuir and Freundlich models were also taken into account, Equations (10) and (11), respectively, and the isotherm parameters obtained with the two fitting models are listed in Table 3.

As can be deduced from the R^2^ values, both the Langmuir and Freundlich isotherms can well explain the adsorption of oleuropein on poly(VI); in particular, from the Langmuir model, we obtained a *Q_max_* value of 306.80 (mg g^−1^), suggesting that poly(VI) is a promising candidate for capturing Oleo in aqueous solution. Nevertheless, the most significant difference concerns to the *Q_max_* values obtained by applying the non-linear and linear forms in the Langmuir fitting. In the case of the linear form of the equation, the *Q_max_* value obtained was 109.7, which is very close to the maximum experimental *Q_e_* value (87 mg g^−1^). However, when applying a non-linear fit, the *Q_max_* value increased significantly to 306.8 mg g^−1^, despite both cases maintaining a high R^2^ value. Such discrepancy most likely results from the non-achievement of adsorption equilibrium under the experimental conditions. This conclusion is supported by the trend in the experimental data, which highlights the high absorption capacity of the material. The efficacy of this macroporous material relies in the synergistic effect between pore sieving, the intrinsic adsorption properties of this class of polymers, the π–π interaction, and the hydrogen bond provided by imidazole moiety. Together, these factors allow for the capture and sequestration of oleuropein with high efficiency. In Table 4 are listed the adsorption performance of similar macroporous adsorbent already reported in the literature for comparison purposes. As can be seen, our outcomes are similar to those previously published, but the main benefit lies in the Oleo’s release from the material, which also occurs in aqueous solvent, as better explained in the next section. This makes the process entirely green in contrast to the organic solvent processes previously reported. 

On the contrary, for Freundlich model fitting, no discrepancies were found between the two forms of the equation, highlighting the strengths of the model.

### 3.4. Release of Oleuropein and Reusability of Poly(VI)

The release of the captured oleuropein from the material is a necessary and fundamental condition to allow for the subsequent use of the molecule in the field of biomedical research and therapeutic development due to the multifaceted role this molecule plays in health and disease prevention. Its strong antioxidant, anti-inflammatory and antimicrobial properties make it a suitable molecule to recover and utilize.

**Figure 5:**
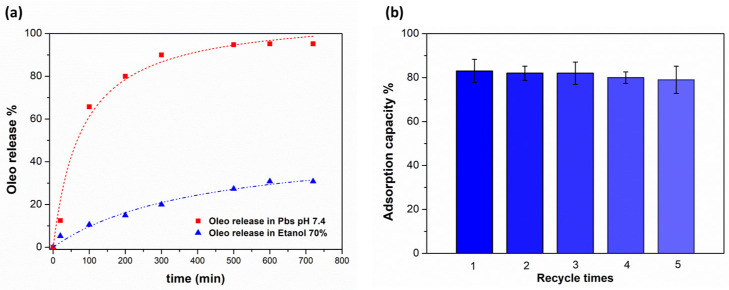
(**a**) Release kinetic of Oleo from poly(VI). (**b**) Adsorption–desorption recycles of Oleo on poly(VI).

In this context, the release of oleuropein from poly(VI) cryogel has been studied, as well as its kinetic release. Considering the high solubility of oleuropein in alcoholic solvents, a solution of ethanol at 70% was added to the poly(VI) cryogel containing 50 ppm of absorbed oleuropein. The release was measured by following the absorbance value at 232 nm at different time points. However, the release percentage in this condition did not exceed 30%, indicating that ethanol is not able to break the interaction of oleuropein with our material (Figure 5a, blue line).

Taking advantage of its ionic strength, PBS (pH 7.4) was selected as a good candidate for oleuropein release. The controlled ionic environment makes PBS suitable for studying release processes that may be influenced by ionic interactions between the polymeric material and the organic molecules. It is well known that high ionic strength solutions can weaken hydrogen bonds due to screening effects, especially in systems where hydrogen-bonding interactions are influenced by electrostatic interactions [34]. As shown in Figure 5a (red line), this eluent was more effective than the ethanolic solution for the adsorbent release, achieving an average desorption rate of 95%. This is consistent with the breaking of hydrogen bonds between oleuropein and the imidazole portion of the polymeric material.

To investigate the reusability of the poly(VI) sorbent, five cycles of adsorption–desorption experiments were carried out under the same conditions described above. Figure 5b demonstrates that the poly(VI) cryogel can be used at least five times without significant loss in adsorption capacity. This result highlights that imidazole-based cryogel exhibits good reusability and could be used to isolate and purify oleuropein from olive leaves.

**Figure 6:**
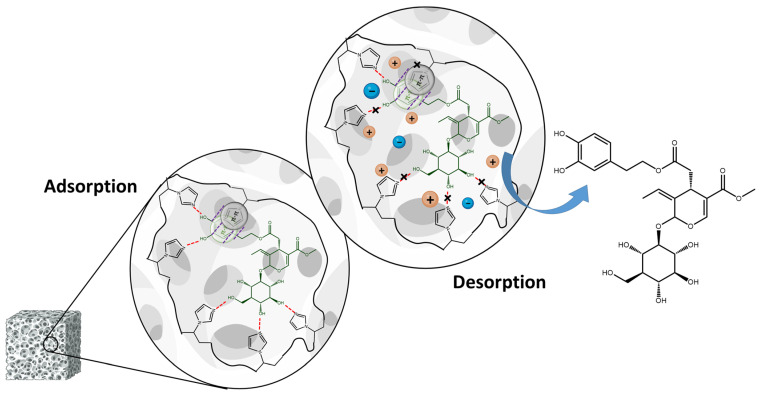
The proposed adsorption mechanism of oleuropein by the hydrogen-bonding and staking interaction with the imidazole moieties of poly(VI) and its desorption in the presence of the PBS with high ionic strength breaking this interaction.

The proposed mechanism of oleuropein adsorption and desorption from poly(VI) is reported in Figure 6.

## 4. Conclusions

A polyvinyl imidazole derivative was synthesized to create a macroporous material with excellent adsorbent properties versus polyphenol compounds. In addition, kinetic and adsorption studies were performed in view of future tests for selective recovery. The choice of the imidazole functional group, renowned for its hydrogen bond acceptor ability and electron-rich aromatic system, was pivotal in integrating these advantageous properties into the polymeric system. This synthesis resulted in a material that exhibits exceptional adsorption capabilities towards oleuropein. Moreover, alteration of the ionic strength facilitates the efficient release of the molecule under sustainable conditions, allowing for the regeneration of the material. Our results highlight the potential of poly(VI) as an efficient tool for recovering this valuable compound through an entirely green process conducted in aqueous solution.

This system provides a low-cost and straightforward method for capturing and releasing oleuropein, effectively addressing a significant gap in the current literature, representing a valid tool for the valorization of oleuropein as well as other phenolic systems, leveraging their health-related advantages, such as antioxidant, antimicrobial, and anti-inflammatory properties.

## Figures and Tables

**Table 1:** Experimental data used for swelling test measurements.

	*W_dry_* (mg)	*W_Swollen_* (mg)
1 replicate	50.2	214.5
2 replicate	50.6	202.5
3 replicate	50.5	209.2

**Table 2:** Kinetic parameters for Oleo on poly(VI).

	Pseudo-First-Order Model		Pseudo-Second-Order Model			
Equation form	*Q_e_* (mg g^−1^)	*K*_1_ (L min^−1^)	R^2^	*Q_e_* (mg g^−1^)	*K*_2_ (g/(mg min^−1^))	R^2^
Non-Linear	8.06	0.030	0.9467	8.96	0.0044	0.9906
Linear	9.56	−2.10 × 10^−6^	0.5378	9.01	0.0054	0.9976

**Table 3:** Adsorption isotherm parameters for the Langmuir and Freundlich models.

	Langmuir Model		Freundlich Model			
Equation form	*Q_max_* (mg g^−1^)	*K_L_* (L min^−1^)	R^2^	1/*n*	*K_F_* (mg g^−1^)/(mL min^−1^)	R^2^
Non-Linear	306.80	6.9 × 10^−4^	0.9972	0.81	0.518	0.9998
Linear	109.76	0.0028	0.9987	0.79	0.55	0.9998

**Table 4:** Adsorption capacity of MARs reported in the literature.

Adsorbent	*Q*_*e*(*exp*)_ (mg g^−1^)	*Q*_(*max*)_ (mg g^−1^) Langmuir Model	References
PS/PVIm	17.98	22.83	[11]
LSA-21	364.72	350.9	[15]
BNKX-5-HP	168.79	262.88	[17]
PS/C-CNT-2	82.59	83.19	[18]

## Data Availability

Data are contained within the article.
